# The Impact of Russia-Ukraine geopolitical conflict on the air quality and toxicological properties of ambient PM_2.5_ in Milan, Italy

**DOI:** 10.1038/s41598-024-55292-2

**Published:** 2024-03-12

**Authors:** Yashar Aghaei, Mohammad Mahdi Badami, Ramin Tohidi, P. S. Ganesh Subramanian, Roberto Boffi, Alessandro Borgini, Cinzia De Marco, Paolo Contiero, Ario Alberto Ruprecht, Vishal Verma, Talal Chatila, Constantinos Sioutas

**Affiliations:** 1 Department of Civil and Environmental Engineering, University of Southern California, 3620 S. Vermont Ave. KAP210, Los Angeles, CA 90089 USA; 2 Department of Civil and Environmental Engineering, University of Illinois at Urbana Champaign, Urbana, IL USA; 3 Fondazione IRCCS, Istituto Nazionale Tumori, Milan, Italy; 4 International Society of Doctors for Environment (ISDE), Arezzo, Italy; 5 Division of Immunology, Boston Children’s Hospital, Harvard Medical School, Boston, MA USA

**Keywords:** Environmental impact, Ecology, Environmental sciences, Engineering

## Abstract

The geopolitical conflict between Russia and Ukraine has disrupted Europe’s natural gas supplies, driving up gas prices and leading to a shift towards biomass for residential heating during colder months. This study assessed the consequent air quality and toxicological impacts in Milan, Italy, focusing on fine particulate matter (PM_2.5_, d_p_ < 2.5 μm) emissions. PM_2.5_ samples were analyzed for their chemical composition and assessed for their oxidative potential using the dithiothreitol (DTT) assay across three periods reflecting residential heating deployment (RHD): pre-RHD, intra-RHD, and post-RHD periods. During the intra-RHD period, PM_2.5_ levels were significantly higher than those in other periods, with concentrations reaching 57.94 ± 7.57 μg/m^3^, indicating a deterioration in air quality. Moreover, levoglucosan was 9.2 times higher during the intra-RHD period compared to the pre-RHD period, correlating with elevated levels of elemental carbon (EC) and polycyclic aromatic hydrocarbons (PAHs). These findings were compared with previous local studies before the conflict, underscoring a significant rise in biomass-related emissions. DTT assay levels during the intra-RHD were 2.1 times higher than those observed during the same period in 2022, strongly correlating with biomass burning emissions. Our findings highlight the necessity for policies to mitigate the indirect health effects of increased biomass burning emissions due to the energy crisis triggered by the geopolitical conflict.

## Introduction

The geopolitical tension between Russia and Ukraine has led to multifaceted effects in various sectors: while political relations and socio-economic aspects are at the forefront^1^, a critical implication demanding attention is the ensuing energy crisis, which poses significant risks to environmental and public health, including the potential exacerbation of air pollution levels^2,3^. Triggered by this energy crisis, the European Union (EU) has faced a substantial disruption in natural gas imports from Russia^4,5^, which escalated the prices of gas across European markets since early 2022^6,7^, leading to a roughly 20% decrease in natural gas consumption^8^. Although the EU has been extensively exploring alternative renewable energy sources with the aim of replacing natural gas^9^, biomass use for heat generation still stands as an economically viable alternative^10^. In line with this shift to biomass, the EU increased its imports by approximately 30% in 2022 compared to the preceding year^8,11^. As Italy has historically relied on both natural gas and biomass for residential energy demands, supported by previous studies on emissions and air quality^12–14^, it is positioned as an important case study for examining the levels and toxicity of atmospheric pollutants in the context of the current geopolitical conflict, which potentially shifts pollution sources.

Since the beginning of 2023, the Italian government has introduced tax reductions on wood pellets, a type of biomass, aimed to decrease residential heating costs and encourage the use of biomass for energy^15^. However, it is important to evaluate the impact of biomass burning on air quality, given that emissions from biomass combustion can contribute significantly to the levels of fine particulate matter (PM_2.5_, particle with aerodynamic diameter < 2.5 μm) in the air during the winter months^16–18^. PM_2.5_ can penetrate deep into the lungs and cause cardiovascular inflammation and lower the overall functions of the respiratory system^19–21^. The International Agency for Research on Cancer (IARC) classified ambient PM_2.5_ as a Group 1 carcinogen for lung cancer^22,23^, and epidemiological studies have also linked long-term exposure to these particles with an increased risk of lung cancer^24–26^. Although the total PM_2.5_ content of the ambient air has been linked to adverse impacts on human health^27,28^, specific chemical components, including carbonaceous species (i.e., organic carbon (OC), levoglucosan, and polycyclic aromatic hydrocarbons (PAHs)), have been consistently associated with the oxidative potential of PM_2.5_^29–31^. Since these toxic components are major PM components of biomass burning emissions^32–34^, evaluation of their oxidative potential is essential for the investigation of the toxicity affecting residents subject to biomass activity emissions^35–37^.

Investigating the toxicity associated with these emissions is critical in areas prone to frequent air pollution episodes, such as the Po Valley in Italy’s Lombardy region, which has experienced degraded air quality over the past few decades, particularly in the winter months^38,39^. One of the primary reasons for the persistent air quality issue is the geographical location of the valley, which is surrounded by the Alps and the Apennines mountains. This topographical situation, combined with stagnant meteorological conditions, limits the effective dispersion of air pollutants emitted in the valley^40–42^. Previous investigations indicated that during the warm seasons, the levels of ambient PM_2.5_ in the valley were mainly influenced by vehicular emissions and the formation of secondary organic aerosols^43,44^, while in the cold seasons, a combination of particular weather conditions and various emission sources, including biomass burning, can lead to severe air pollution episodes^45,46^. The ongoing energy crisis, driven by tensions between Russia and Ukraine, poses the risk of exacerbating the situation by possibly increasing the contribution of residential biomass burning as a source of pollution in the region. In addition to adverse impacts on air quality and public health, biomass burning contributes to climate change by emitting greenhouse gases and black carbon, influencing global warming through changes in solar radiative forces^47,48^.

This study aimed to determine potential changes in ambient PM_2.5_ chemical components impacting air quality and associated toxicity properties due to the geopolitical tensions between Russia and Ukraine. Ambient PM_2.5_ samples were collected within three distinct periods with consideration of the influence of residential heating deployment (RHD), including pre-RHD, intra-RHD, and post-RHD periods. The collected samples were analyzed for chemical species, and the dithiothreitol (DTT) assay was utilized to determine the oxidative potential of these samples. The levels of PM_2.5_ components and the associated toxicological characteristics were compared with previous investigations in that area conducted during winter before the conflict, enabling the observation of atmospheric pollutants’ levels regardless of variations in meteorological factors from winter (i.e., intra-RHD) to other seasons (i.e., pre-RHD and post-RHD periods).

## Methods

### Sampling site and period

Sample collection was carried out at Bareggio, a Milan suburb area located 14 km northwest subjected to temperature variations, during three specific periods: pre-RHD period (late-October–early-November), intra-RHD period (late-November–February), and post-RHD period (mid-March–early-April) in 2022 and 2023. The region’s rich historical air quality data^12–14^, long-standing history of biomass use for residential heating^43,44^, and unique topographical location^40–42^, alongside the energy policy changes^15^, driven by recent geopolitical tension between Russia and Ukraine, presented Milan as a prime case study for investigating the impacts of conflict on air quality. The meteorological conditions (i.e., temperature, relative humidity, and wind speed) within the sampling period were obtained from the closest meteorological station, the Roveda di Sedriano (MI) site in Bareggio, presented in Table S1. Low wind speed and relatively stable temperature further illustrated atmospheric conditions favoring pollutant accumulation, which were in agreement with previous studies documenting the Po Valley’s stable atmospheric conditions, influencing air quality, especially in terms of PM_2.5_ concentration^49,50^.

A total of 17 ambient PM_2.5_ samples were collected on pre-baked quartz filters (37 mm, Pall Life Sciences, 2-μm pore size, Ann Arbor, MI, USA) for weekly periods, utilizing two Sioutas Personal Cascade Impactor Samplers (PCISs, SKC Inc., Eighty-Four, PA, USA), each operating with a flow rate set at 9 L per minute^51,52^. The collected mass of PM_2.5_ was determined as the weight differential between the filters before and after sampling, following an equilibration process under controlled laboratory conditions, temperature range of 22–24 °C, and relative humidity range of 40–50%. This weight measurement was conducted using a microbalance (MT5, Mettler Toledo Inc., Columbus, OH, USA) with an accuracy level of ± 0.001 mg. Further details about the sampling procedure were provided in the supplementary information section.

### Chemical and toxicological analysis

The collected samples were subjected to chemical analysis at the Desert Research Institute (DRI) to determine their organic carbon (OC) and elemental carbon (EC) content, levoglucosan, polycyclic aromatic hydrocarbons (PAHs), water-soluble ions, as well as total metals. The measurement of OC and EC, as well as the assessment of the volatility of OC fractions, were conducted using a thermal/optical method^53^. Gas chromatography/mass spectrometry (GC/MS) was utilized to measure levoglucosan and other specific organic compounds^54^. Furthermore, ion chromatography (IC) and inductively coupled plasma mass spectroscopy (ICP-MS) were employed to determine the concentrations of water-soluble inorganic ions as well as metals and trace elements^55,56^, respectively. The chemical components in the analysis included OC, EC, crustal metal and trace elements, and water-soluble inorganic ions (i.e., NH_4_^+^ SO_4_^3−^, NO_3_^−^, PO_4_^3−^, Cl^−^, Na^+^, and K^+^). To convert OC to organic matter (OM), we used a conversion factor of 2.0 for the intra-RHD period and 1.8 for the pre- and post-RHD periods^57^. For the evaluation of the oxidative potential, the DTT assay was employed by the Illinois Lab for Aerosol Research at the University of Illinois Urbana-Champaign, a widely used method for this purpose^58–61^. The oxidative potential represents the capacity of PM to produce reactive oxygen species that can lead to oxidative stress, potentially resulting in adverse health outcomes^62^. In the DTT assay, the consumption rate of DTT reflects the oxidative potential of the PM samples, as it measures the decline in cellular antioxidants due to their conversion to their disulfide states^63^. Previous studies have identified various PM constituents, including PAHs and metals like Fe, Cu, Ni, Mn, and Zn, as contributing factors to the rate of DTT depletion^64,65^. In this study, collected samples on quartz filter extracted in methanol were filtered in a cell-free system, and the linear rate of DTT depletion was determined per time unit. Further details about the operation of the DTT assay are available in earlier literature^62,66^. The rate of DTT consumption was normalized to the volume of the air sampled, obtaining intrinsic oxidative potentials measured in the unit of nmol/(min m^3^). Further details about chemical and toxicological analyses, as well as quality assurance and control, were provided in the supplementary information section.

## Results and discussion

### Total mass concentrations

**Figure 1:**
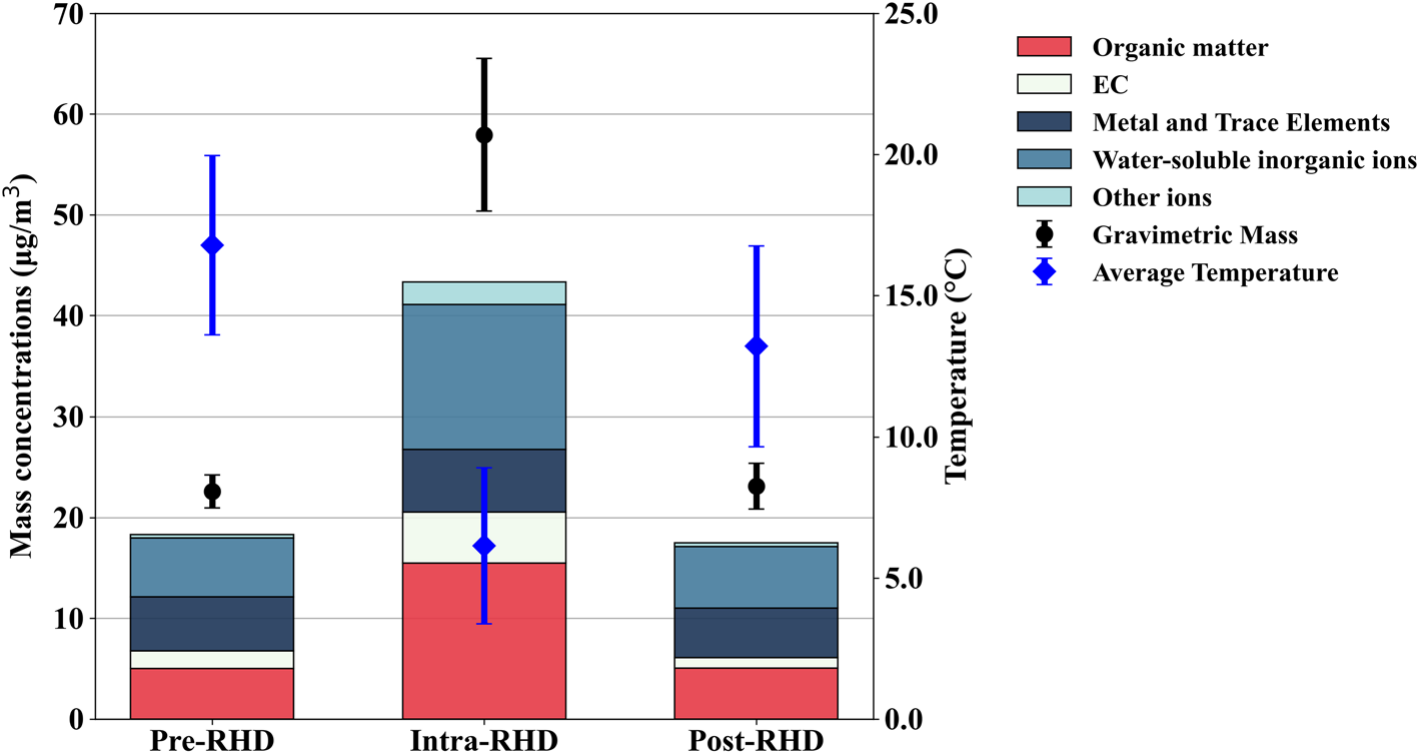
Mass concentrations of the collected PM_2.5_ samples with their chemical composition and average temperature across different measurement periods (i.e., pre-, intra-, and post-RHD). Error bars on the graphs represent the standard deviation, indicating the variability for average temperature and gravimetric mass of the PM_2.5_ samples.

Figure 1 shows the mass concentrations of the collected PM_2.5_ in samples with contributed chemical components and average ambient temperatures during measurement periods to observe the influence of domestic heating deployment.

As shown in Fig. 1, the concentration of PM_2.5_ was significantly higher during the intra-RHD period, averaging 57.94 ± 7.57 μg/m^3^, compared to 22.61 ± 1.64 and 23.13 ± 2.27 μg/m^3^ in the pre- and post-RHD periods. A significant increase in the mass concentration of total PM_2.5_ was observed during the intra-RHD period for a range of constituents, including OM, EC, crustal metals and trace elements, and water-soluble ions (i.e., NH_4_^+^ SO_4_^2−^, NO_3_^−^, PO_4_^3−^, Cl^−^, Na^+^, and K^+^) with recorded values as 16.56, 5.50, 6.32, and 16.46 μg/m^3^, respectively. However, average PM_2.5_ mass concentrations during wintertime in 2020 were reported to be 42.63 ± 16.47 μg/m^3^, which was lower than our reported PM_2.5_ concentration. While lower temperatures and restricted atmospheric mixing height contribute to elevated pollutant levels^49,50^, the significant difference in PM_2.5_ concentration between the cold months of 2023 and 2020 shows an exacerbating air quality condition, likely due to changes in emission sources contributing to the elevated PM_2.5_ levels, which will be explored in the following sections through the analysis of PM_2.5_ chemical components.

### Trace elements and metals

**Figure 2:**
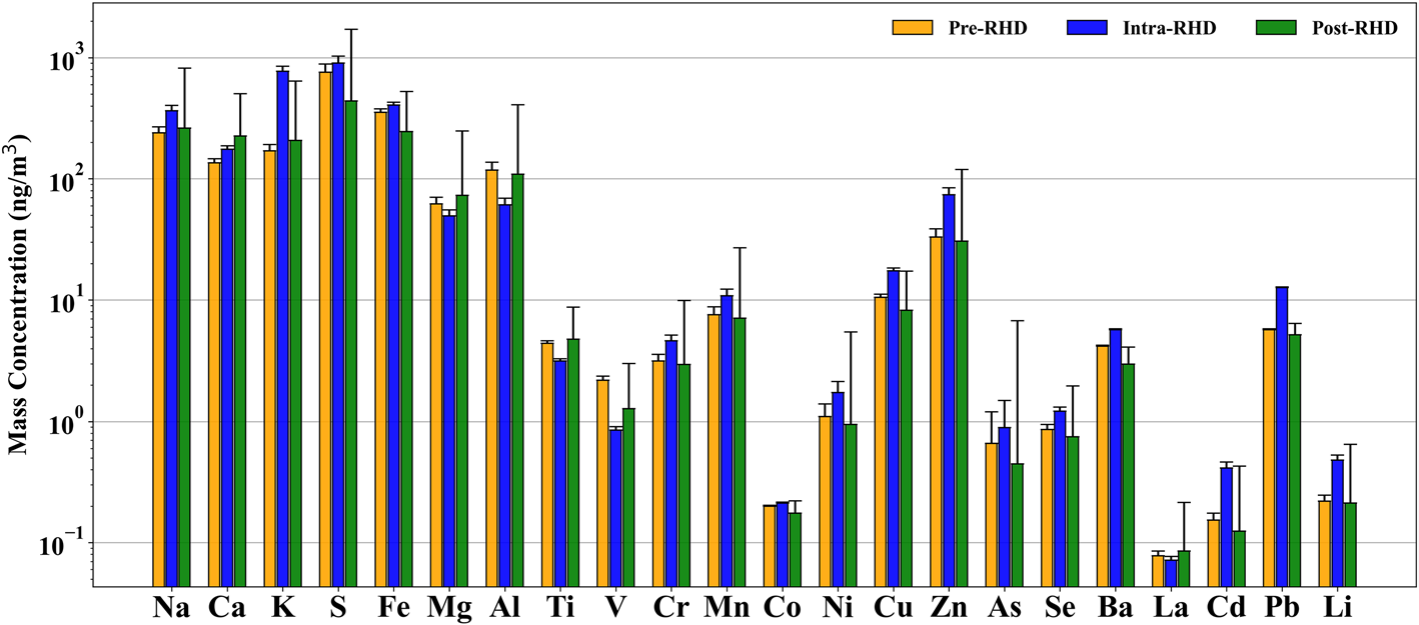
Average concentrations of trace elements and transition metals across different measurement periods (i.e., pre-, intra-, and post-RHD). Error bars on the graphs represent the Standard deviation, indicating the variability for the concentration of each element in the PM_2.5_ samples.

The average concentrations of trace elements and transition metals during the pre-, intra-, and post-RHD periods in our sampling location are presented in Fig. 2. The chemical speciation commonly associated with industrial emissions (e.g., Se, As, Cd, Pb, and V)^68–70^, as well as mineral and road dust emissions (e.g., Fe, Li, Cr, Mn, Ni, Cu, Zn, and Ba)^71–73^, showed higher levels during the intra-RHD period, which were approximately between 1.5 and 1.7 times higher than pre- and post-RHD periods. In the Po Valley, the majority of PM_2.5_-bound metals and trace elements primarily originate from anthropogenic sources (e.g., vehicle emissions and industrial operations)^43,44,74^, which have relatively stable emissions throughout the year^46^, so the temporal variations of these emitted species are influenced mainly by seasonal weather conditions^42,50^.

The mass concentration of K, a biomass burning chemical marker^75^, increased during the intra-RHD period to 771.86 ng/m^3^, with concentrations of 4.5 and 3.8 times higher than the pre- and post-RHD periods. This significant rise in K mass concentration cannot be solely explained by meteorological conditions. The increased K values were potentially attributed to elevated biomass burning emissions within the intra-RHD period when people switched from using natural gas to biomass for residential heating purposes, which could be justified by the significant difference between the ratio of the intra-RHD period to the other periods.

### Carbonaceous components

**Figure 3:**
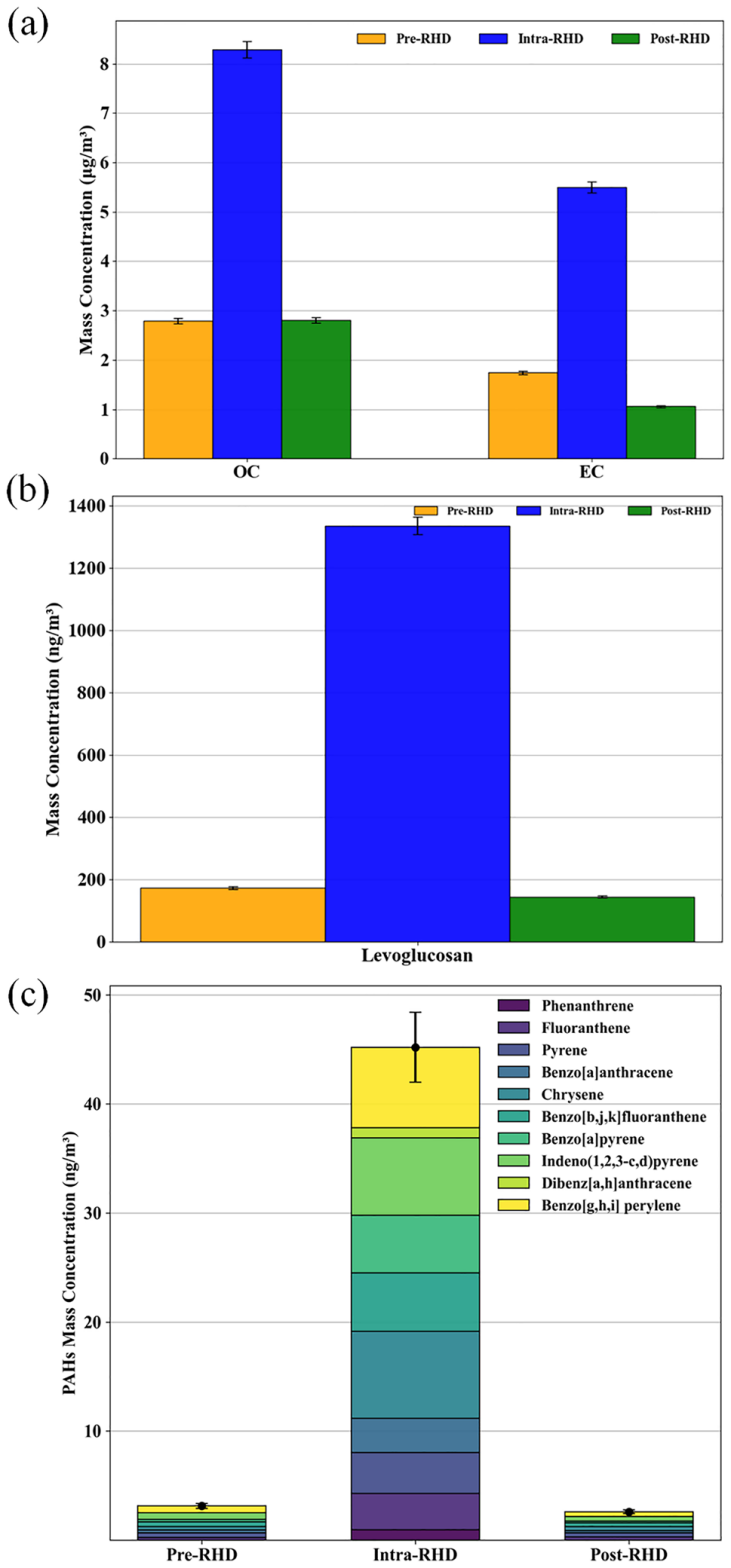
Average concentrations of (**a**) OC and EC, (**b**) levoglucosan, and (**c**) individual PAH species across different measurement periods (i.e., pre-, intra-, and post-RHD). Error bars on the graphs represent the standard deviation, indicating the variability for the concentration of each component in the PM_2.5_ samples.

Figure 3 displays the average concentrations of carbonaceous components over the pre-, intra-, and post-RHD periods. Both OC and EC displayed higher mass concentrations during the intra-RHD period, with values reported as 8.28 and 5.50 μg/m^3^, respectively. The OC concentration during the intra-RHD period was approximately 2.9 times higher than those observed during both the pre- and post-RHD periods. Similarly, the EC level increased, reaching 3.2 times higher compared to the pre-RHD period and 5.3 times higher than the post-RHD period (see Fig. 3a).

Levoglucosan, which has been used as a tracer of biomass burning^76^, exhibited increased value during the intra-RHD period, with a concentration of 1335.0 ng/m^3^. The levoglucosan level during the intra-RHD period was 7.7 times higher than those observed during the pre-RHD period and 9.2 times higher than the post-RHD period.

PAHs are carbonaceous components consisting of multiple aromatic rings produced during the incomplete combustion of fossil fuels and biomass burning^77,78^. During the intra-RHD period, the PAHs levels showed a similar pattern, with an increased concentration of 45.18 ng/m^3^, which was 14.5 and 17.5 times higher than the pre- and post-RHD periods, respectively. Among individual PAHs, chrysene, pyrene, benzo[a]pyrene, and benzo[ghi]perylene are found to be particularly abundant in biomass burning emissions^45,79^, which showed higher levels during the intra-RHD period compared to the two other periods. Chrysene and pyrene showed an approximate 25 and ninefold increase from 0.31 and 0.41 ng/m^3^ in the pre-RHD to 7.98 and 3.74 ng/m^3^ during the intra-RHD period, respectively, and these values decreased to 0.35 and 0.38 ng/m^3^ in the post-RHD period. Similarly, benzo[a]pyrene and benzo[ghi]perylene showed higher concentrations during the intra-RHD period, with values reported as 5.26 and 7.36 ng/m^3^, respectively, which are 22 and 12 times higher than those recorded during the pre-RHD period; subsequently, these values declined to 0.17 and 0.42 ng/m^3^ in the post-RHD period.

Although increased levels of the carbonaceous species can be partially attributed to more stable atmospheric wintertime conditions^50,80^, which limit effective dispersion in the atmosphere^46,81^, this alone cannot fully account for the observed high concentrations. During the intra-RHD period, the elevated concentrations of biomass burning tracers, including levoglucosan and PAHs, along with the strong linear correlations observed between levoglucosan and total PAHs (R^2^ = 0.95, *p*-value < 0.001), as well as EC (R^2^ = 0.97, *p*-value < 0.001), demonstrate the significant impact of biomass burning on the rise in carbonaceous component levels.

A comparison of our reported carbonaceous species concentrations with earlier studies in this region during cold months is presented in Table 1. In our study, the concentration of PM_2.5_-bound OC, a marker for both traffic and biomass burning emissions, was in a comparable range with PM_10_-bound OC measurements from several locations across the Po Valley^16,67^. Additionally, the OC levels reported in our study were higher than those in other studies focusing on ambient PM_2.5_^43,44,82^. Similarly, the EC level measured during the intra-RHD period was higher than previously recorded values across the region for the same time span in past years, highlighting the effect of increased domestic biomass burning during the ongoing conflict. The levoglucosan mass concentration observed in our study was significantly higher than the previously reported values at urban/rural locations in Po Valley^16,43,44,67,82^. The concentrations of PAHs measured during the intra-RHD period were also notably higher than those in urban and suburban regions of Milan^16,43,44,67,82^, and even exceeded levels found on sites in Beijing, Wuhan, and Shanghai with the average value of 34.37 ng/m^3^ ^83^. Moreover, the PAHs levels in our study were comparable to those in polluted urban/suburban areas in the Czech Republic, with an average of 39.25 ng/m^3^^84^. Additionally, Table 1 shows a comparison between three distinct periods: normal life and traffic patterns in 2020 (January–February), the first lockdown period of COVID-19 in 2020 (March), and our study during the intra-RHD period in 2023, affected by the ongoing conflict. There was a noticeable decrease in the levels of carbonaceous components during the pandemic^82^ compared to the pre-pandemic period^16^, a reduction that is likely attributable to decreased human activity and traffic at that time. Our findings showed higher levels of EC, levoglucosan, and PAHs during the intra-RHD period compared to those reported during the pre-pandemic by Pietrogrande et al.^16^. Considering that EC can also be a tracer of traffic emissions, we conducted further analysis to determine whether traffic might be contributing to its increased levels. Table 2 displays data from the Environmental Protection Agency of Lombardy (Agenzia Regionale per la Protezione Ambientale (ARPA)) website for Milan—via Pascal air quality station on specific markers for traffic emissions (i.e., NO_2_ and C_6_H_6_)^85–87^ and contrasts these levels during the measurement periods of our study and the study conducted by Pietrogrande et al.^16^.

**Table 1:** Comparison of biomass burning tracers during the winter months in the region.

Study	Year	Sample	Species			
			OC (μg/m^3^)	EC (μg/m^3^)	PAHs (ng/m^3^)	Levoglucosan (ng/m^3^)
Current study	2023	PM_2.5_	8.28 ± 0.16	5.50 ± 0.11	45.18 ± 3.27	1335.0 ± 28.25
Perrone et al.^44^	2006–09	PM_2.5_	6.04	–	25.8 ± 4.3	502 ± 134
Altuwayjiri et al.^82^	2020	PM_2.5_	2.41 ± 0.14	0.28 ± 0.03	1.42 ± 0.21	53.85 ± 8.06
Daher et al.^43^	2009–10	PM_10_	11.65	0.75	7.47	53
Pietrogrande et al.^16^	2020	PM_10_	10.38 ± 4.23	1.64 ± 0.9	3.71 ± 3.77	1100 ± 710
Pietrogrande et al.^67^	2020	PM_10_	11.10 ± 4.56	1.47 ± 0.83	2.82 ± 2.22	1070 ± 720

**Table 2:** Comparison of traffic-related emissions during the winter months.

Month/year	NO_2_ (μg/m^3^)		C_6_H_6_ (μg/m^3^)	
	2023	2020	2023	2020
January	40.74	56.07	2.49	3.8
February	40.47	44.83	1.96	2.01

Both NO_2_ and C_6_H_6_ levels were slightly lower in 2023 compared to the same timeframe in 2020 (*p*-value < 0.05), suggesting a small decrease in traffic-related emissions in 2023. As a result, the high levels of EC observed in our study suggest an alternative emission source rather than traffic emissions significantly contributes to atmospheric pollutants. Given that traffic emissions during our 2023 measurement period were comparable to pre-pandemic levels in 2020, the rise in the levels of carbonaceous compounds in our study aligns with Italy’s tax reduction for biomass, suggesting these policies have boosted biomass burning for residential heating.

### Oxidative potential of PM_2.5_

**Figure 4:**
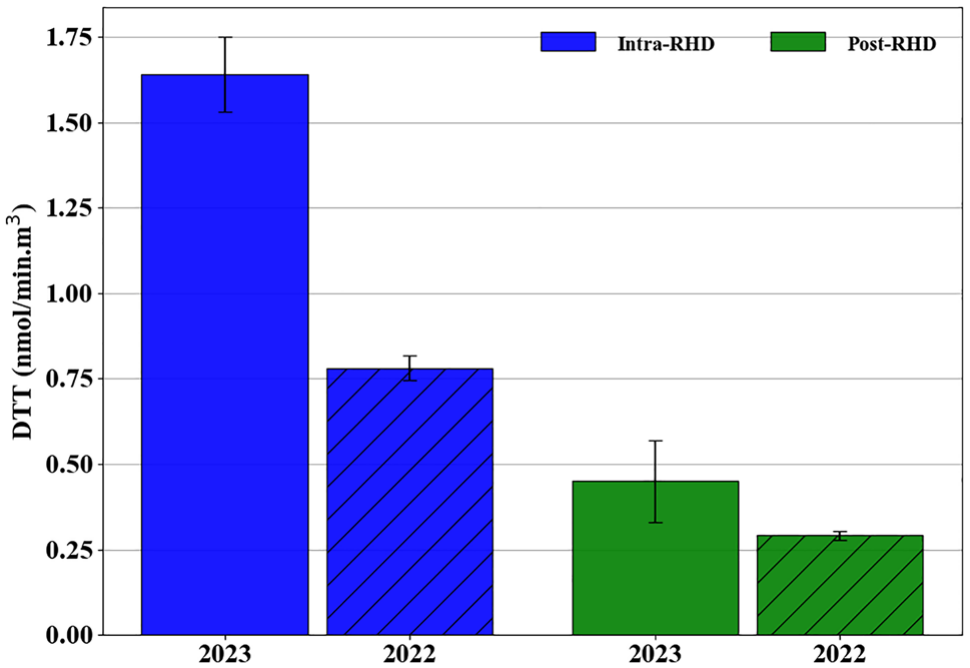
PM_2.5_ oxidative potential as measured by the DTT assay across intra- and post-RHD periods in 2022 and 2023. Error bars on the graphs represent the standard deviation, indicating the variability for the DTT assay in the PM_2.5_ samples.

Previous research conducted in the Po Valley indicated that the oxidative potential of PM is mainly driven by certain metals (e.g., Cr, Mn, Fe, Ni, and Cu), as well as carbonaceous compounds such as OC, levoglucosan, and PAHs^59,88,89^. Figure 4 shows the results of PM_2.5_ oxidative potential as measured by the DTT assay of collected samples. During the intra-RHD period in 2023, with the significant increase of biomass burning tracers in PM_2.5_ composition (i.e., levoglucosan and PAHs), the oxidative potential measured by DTT assay showed an elevated level of 1.64 nmol/(min m^3^), which was 3.6 times higher than those observed during the post-RHD in 2023. To further analyze the toxicity of PM_2.5_ emissions, the oxidative potential of collected samples during the intra-RHD period in the preceding year was evaluated. During the intra-RHD period in 2022, DTT analysis showed a higher value of 0.78 nmol/(min m^3^), which was 2.6 times higher than those observed during the post-RHD in 2022, which is in line with the trend we observed in 2023. One factor that could increase the oxidative potential is stable atmospheric conditions prevailing in the winter, leading to the accumulation and aging of particles in the atmosphere^42,44,90^, thereby amplifying their oxidative potential and, consequently, their toxicity. However, for the intra-RHD periods, the DTT activity in 2023 was 2.1 times higher than the levels observed in 2022, indicating increased oxidative potential in PM_2.5_ samples. For the post-RHD periods, the DTT findings were comparable in both years, with observed values of 0.45 and 0.29 nmol/(min m^3^) in 2023 and 2022, respectively. While the measured DTT analysis during the post-RHD was consistent with findings in both years, the result during the intra-RHD period in 2023 was notably higher in contrast to the previous year, suggesting that the high-density emissions from biomass burning could be another key factor in increasing the oxidative potential of ambient PM_2.5_.

In Table 3, we compared levels of DTT activity in the current study to data from earlier studies in the same area during the winter before the geopolitical tensions. The oxidative potential measured by the DTT assay reported in 2023 was significantly higher than both our measurements in 2022 and previous investigations^16,82,91^. Despite a slight reduction in road traffic, the findings indicated that the PM_2.5_ toxicity in the urban background site of the Bareggio is drastically increased compared to previous investigations in the region, suggesting that biomass combustion emissions rather than road traffic are possibly contributing significantly to elevated oxidative potential. These observations align with increased mass concentrations of the collected PM_2.5_ and raised concentrations of biomass combustion markers in the intra-RHD period, as covered in previous sections.

**Table 3:** Comparison of DTT activity during the winter months in the region.

Study	Year	Samples	DTT nmol/(min m^3^)
Current study	2023	PM_2.5_	1.64 ± 0.11
Current study	2022	PM_2.5_	0.78 ± 0.03
Altuwayjiri et al.^82^	2020	PM_2.5_	0.73 ± 0.13
Simonetti et al.^91^	2017	PM_10_	0.07 ± 0.01
Pietrogrande et al.^16^	2020	PM_10_	0.62 ± 0.15
Pietrogrande et al.^67^	2020	PM_10_	0.39 ± 0.15

To better understand the influence of residential biomass burning emissions on the oxidative potential of PM_2.5_, we conducted a statistical analysis to determine the correlation between DTT activity and the mass concentrations of specific chemical PM_2.5_ compounds. Table 4 presents the findings from a Spearman rank regression analysis, which explored the relationship between DTT activity and the chemical compounds. Our analysis showed strong correlations between DTT activity levels and carbonaceous compounds, such as EC, levoglucosan, and PAHs (*p*-values < 0.05), highlighting the importance of biomass burning tracers in the oxidative potential and toxicity of PM_2.5_ particles. While biomass combustion markers were highly correlated with the DTT activity, trace elements (e.g., Se, Pb, V, Fe, Li, Cr, Mn, Ni, Cu, Zn, and Ba) and water-soluble inorganic ions (e.g., NH_4_^+^ SO_4_^3−^, NO_3_^−^) showed relatively weak correlations. The strong correlation between biomass burning tracers and the oxidative potential of PM_2.5_ further corroborates the major role of biomass burning in the adverse indirect health impacts. It should be noted that epidemiological studies have linked exposure to PM_2.5_-bound OC and EC to increased cardiovascular disease admissions, heightened inflammatory responses, blood pressure changes, and respiratory inflammation^92–95^. PAH was also associated with increased inflammatory markers such as interleukin-6, indicating potential respiratory and systemic inflammation^96^, highlighting the significant health risks posed by geopolitical tensions between Russia and Ukraine.

**Table 4:** Spearman correlation coefficients between the DTT activity (nmol/(min m^3^) air) and mass concentration (μg/m^3^) of different chemical species.

Species	DTT activity		Species	DTT activity	
	R	*P*-value		R	*P*-value
PAHs	**0.88**	**0.01**	Li	0.14	0.78
Levoglucosan	**0.77**	**0.05**	Cr	0.42	0.39
EC	**0.82**	**0.03**	Mn	0.20	0.70
OC	**0.71**	**0.10**	Ni	0.42	0.39
K	**0.94**	**0.004**	Cu	0.48	0.32
As	**0.82**	**0.03**	Zn	0.25	0.62
Cd	**0.77**	**0.05**	Ba	0.60	0.20
Pb	0.60	0.20	NH_4_^+^	− 0.31	0.54
V	− 0.65	0.15	SO_4_^2−^	− 0.37	0.46
Fe	0.20	0.70	NO_3_^−^	0.25	0.62

Values in Bold indicate statistically significant correlations.

## Conclusions

This study aimed to assess the concentrations of ambient PM_2.5_ and its constituents, as well as the oxidative potential in Milan, Italy, in response to the geopolitical tensions between Russia and Ukraine, which likely prompted a shift to biomass-based residential heating during colder months. To evaluate the effects of this change in heating sources on air pollution, PM_2.5_ samples were collected in the suburban region of Milan using two PCISs during cold and warm periods to consider the effect of residential heating deployment: pre-, intra-, and post-RHD periods. Our findings indicated a substantial rise in PM_2.5_ levels and biomass burning markers, such as K, levoglucosan, PAHs, and EC, during the intra-RHD period, which were among the highest levels reported in prior studies conducted regionally. Of particular note, the PM_2.5_ oxidative potential increased up to 2 times during the intra-RHD period, as opposed to the levels reported in recent studies in the area. Our statistical analysis showed that biomass burning tracers had significant correlations with elevated DTT activity, indicating potential health hazards associated with changes in heating practices due to energy considerations. Furthermore, our study highlights the considerable impact of biomass combustion markers on PM_2.5_ concentration and toxicity within Milan’s metropolitan area, necessitating policy action to mitigate these emissions and their harmful indirect health effects. Research into cleaner biomass technologies, combined with policy incentives for renewable energy adoption such as wind power, alongside the updating of building standards for higher energy efficiency, can significantly contribute to improvements in air quality and public health.

### Supplementary Information

Supplementary Information.

## Data Availability

All data used in this paper are available from the authors upon request.
