# Supplementary material for: The Impact of Russia-Ukraine geopolitical conflict on the air quality and toxicological properties of ambient PM2.5 in Milan, Italy
Source: Sci Rep. 2024 Mar 12;14:5996. doi: 10.1038/s41598-024-55292-2 (PMC10933473; doi:10.1038/s41598-024-55292-2)
Supplement: Supplementary file 1 — Supplementary Information. [file 41598_2024_55292_MOESM1_ESM.pdf]

Supplementary Information of

The Impact of Russia-Ukraine geopolitical conflict on the air quality and toxicological properties of ambient $PM_{2.5}$ in Milan, Italy.

Yashar Aghaei[1], Mohammad Mahdi Badami[1], Ramin Tohidi[1], P.S. Ganesh Subramanian[2], Roberto Boffi[3], Alessandro Borgini[3], Cinzia De Marco[3], Paolo Contiero[3], Ario Alberto Ruprecht[3,4], Vishal Verma[2], Talal Chatila[5], Constantinos Sioutas[1*]

[1]*University of Southern California, Department of Civil and Environmental Engineering, Los Angeles, California, USA*

[2]*University of Illinois at Urbana Champaign, Department of Civil and Environmental Engineering, Urbana, IL, USA*

[3]*Fondazione IRCCS, Istituto Nazionale Tumori, Milan, Italy*

[4]*International Society of Doctors for Environment (ISDE), Italy*

[5]*Harvard Medical School, Division of Immunology, Boston Children's Hospital, Boston, MA USA*

*Corresponding author:

Constantinos Sioutas, Sc.D.

Fred Champion Professor, Civil and Environmental Engineering

University of Southern California

3620 S. Vermont Ave. KAP210, Los Angeles, CA 90089

E-mail: sioutas@usc.edu

Telephone: 213-740-6134

Table S1. Variation (Mean ± SD) of meteorological data for different periods.

**Period**	**Temperature (°C)**	**Wind speed (km/h)**
Pre-RHD	16.76 ±3.17	6.21 ±2.51
Intra-RHD	6.23 ±2.71	4.97 ±2.39
Post-RHD	13.16 ±3.54	8.94 ±4.14

Quality assurance and control

Sampling procedure

In this study, meticulous attention was given to the calibration and accuracy of PCISs to ensure the reliability of our data. Standard operating procedures included flow rate verifications and leak tests, ensuring samplers adhered to manufacturer specifications for precise $PM_{2.5}$ collection, which was detailed in prior studies[1–9]. Upon passing acceptance tests, samplers were deployed in the monitoring site, where recalibration and regular performance checks were conducted according to fixed schedules. The quartz filters used for sampling were prebaked, subsequently stored in freezers in Teflon petri dish containers lined with pre-fired aluminum foil, and then equilibrated under controlled conditions to avoid carbon contamination. Additionally, Polonium-210 sources were used for neutralization before weighing with an MT5 microbalance (Mettler-Toledo Inc., Columbus, OH; uncertainty of 1 μg). Moreover, Sterile, solvent-rinsed stainless-steel tweezers were used for all the work with respect to filters. To maintain samples integrity, our team followed chain-of-custody procedures to maintain sample integrity, including the preparation of filter packs, all carefully labeled and shipped in dry ice to the field site in a sealed cooler to prevent any contamination. The concentrations of collected samples were calculated as a function of sample volumes, calculated from flow rates and sampling durations. Cross-

verification of $PM_{2.5}$ mass concentrations with the Roveda di Sedriano (MI) air monitoring site in Bareggio and maintaining a minimum of 85% successful data capture rate further underscored the robustness of our data collection process. This comprehensive approach to calibration, accuracy, and data handling was pivotal in ensuring the reliability of the chemical and DTT assay analyses conducted at facilities of the DRI and University of Illinois Urbana-Champaign (UIUC), respectively.

Chemical and toxicological analysis

The measurement of OC and EC, as well as the assessment of the volatility of OC fractions, were conducted using a multiwavelength thermal/optical carbon analyzer (Magee Scientific, Berkeley, CA, USA), adhering to the protocols delineated by the interagency monitoring of protected visual environments (IMPROVE_A)[10]. The protocol involved a gradual heating of the quartz filter to specific temperatures for the determination of OC concentrations, followed by a temperature increase for EC level assessment. Additionally, gas chromatography-mass spectrometry instruments (GC-MS, Model 6890N GC/5973 MS detectors, Agilent, Santa Clara, CA, USA) were utilized to measure levoglucosan and PAH[11]. Filters were extracted with methanol and methylene chloride using sonication and then concentrated using a rotary evaporator and then a nitrogen evaporator. The extract was derivatized using diazomethane, which converted carboxylic acids to methyl esters for the quantification of PAH[12]. A second derivatization was also performed using N,O-bis-(trimethylsilyl)trifluoroacetamide and 1% Trimethylchlorosilane, which silylated hydroxyl substituents and allowed levoglucosan quantification[13]. Moreover, additional information on sample processing (e.g., handling, extraction methods, digestion) and details of the analytical procedures used in GC-MS were provided in previous studies[12–14]. Furthermore, the measurement of inorganic ion content was

conducted through ion chromatography (IC), a method involving the extraction of particles into ultrapure deionized water using sonication, followed by filtration of the resultant solution and subsequent quantification of ion concentrations, as detailed in previous studies[15,16]. The inductively coupled plasma mass spectroscopy (ICP-MS) was employed to determine the concentrations of metals and trace elements, involving a hot block acid digestion process to extract particles from the filter into an acidic solution containing nitric (HNO3), hydrochloric (HCl), and hydrofluoric (HF) acids, as elaborated in previous studies[17,18]. After digestion, the samples were diluted with deionized water, aerosolized, and then introduced into the ICP-MS instrument (Thermo Finnigan Element2, Thermo Fisher Scientific Inc., Waltham, MA, USA). Furthermore, DRI's Environmental Analysis Facility (EAF) operations adhere to stringent quality control and assurance protocols[19], and the facility is accredited by the Texas Commission on Environmental Quality (TCEQ) through the National Environmental Laboratory Accreditation Program (NELAP)[20,21], and US Environmental Protection Agency (EPA)[22–24].

The DTT analysis used high-purity reagents with stringent storage and handling protocols. This analysis, conducted with an automated system, included thorough calibration and validation[25]. The reaction vial (RV) was placed inside a thermomixer (550 rpm, 37 ^{0}C) and then mixed with 5,5’-dithiobis-2-nitrobenzoinc acid (DTNB), followed by dilution with DI water in conical centrifuge vial (measurement vial, MV)[26]. DTT was quantified by measuring the absorption of the diluted MV mixture using a Liquid Waveguide Capillary Cell (LWCC-M-100; World Precision Instruments, Sarasota, FL, USA) connected to a spectrophotometer (Ocean Optics, Orlando, FL, USA) containing an ultraviolet-visible light source (Ocean Optics, Orlando, FL, USA). It should be noted for data acquired from laboratory analyses of chemical species and

DTT assay, averages and standard deviations of field blank measurements were calculated and subtracted from measurements of chemical species and oxidative potential.

All calculations, statistical and analytical methods, including regression models and data visualizations, were performed using Python.

References

1. Singh, M., Misra, C. & Sioutas, C. Field evaluation of a personal cascade impactor sampler (PCIS). *Atmos Environ* **37**, 4781–4793 (2003).

2. Kam, W., Cheung, K., Daher, N. & Sioutas, C. Particulate matter (PM) concentrations in underground and ground-level rail systems of the Los Angeles Metro. *Atmos Environ* **45**, 1506–1516 (2011).

3. Pakbin, P., Hudda, N., Cheung, K. L., Moore, K. F. & Sioutas, C. Spatial and Temporal Variability of Coarse (PM10−2.5) Particulate Matter Concentrations in the Los Angeles Area. *Aerosol Science and Technology* **44**, 514–525 (2010).

4. Jalali Farahani, V., Altuwayjiri, A., Taghvaee, S. & Sioutas, C. Tailpipe and Nontailpipe Emission Factors and Source Contributions of PM10on Major Freeways in the Los Angeles Basin. *Environ Sci Technol* **56**, 7029–7039 (2022).

5. Aldekheel, M., Farahani, V. J., Tohidi, R., Altuwayjiri, A. & Sioutas, C. Development and performance evaluation of a two-stage cascade impactor equipped with gelatin filter substrates for the collection of multi-sized particulate matter. *Atmos Environ* **294**, 119493 (2023).

6. Badami, M. M., Tohidi, R., Jalali Farahani, V. & Sioutas, C. Size-segregated source identification of water-soluble and water-insoluble metals and trace elements of coarse and fine PM in central Los Angeles. *Atmos Environ* **310**, 119984 (2023).

7. Shirmohammadi, F. *et al.* Oxidative potential of on-road fine particulate matter (PM2.5) measured on major freeways of Los Angeles, CA, and a 10-year comparison with earlier roadside studies. *Atmos Environ* **148**, 102–114 (2017).

8. Aldekheel, M. *et al.* Identifying urban emission sources and their contribution to the oxidative potential of fine particulate matter (PM2.5) in Kuwait. *Environmental Pollution* **343**, 123165 (2024).

9. Misra, C., Singh, M., Shen, S., Sioutas, C. & Hall, P. M. Development and evaluation of a personal cascade impactor sampler (PCIS). *J Aerosol Sci* **33**, 1027–1047 (2002).

10. Chow, J. C. *et al.* The IMPROVE_A temperature protocol for thermal/optical carbon analysis: Maintaining consistency with a long-term database. *J Air Waste Manage Assoc* **57**, 1014–1023 (2007).

11. Schauer, J. J., Kleeman, M. J., Cass, G. R. & Simoneit, B. R. T. Measurement of emissions from air pollution sources. 2. C1 through C30 organic compounds from medium duty diesel trucks. *Environ Sci Technol* **33**, 1578–1587 (1999).

12. Schauer, J. J., Kleeman, M. J., Cass, G. R. & Simoneit, B. R. T. Measurement of Emissions from Air Pollution Sources. 5. C1−C32 Organic Compounds from Gasoline-Powered Motor Vehicles. *Environ Sci Technol* **36**, 1169–1180 (2002).

13. Nolte, C. G., Schauer, J. J., Cass, G. R. & Simoneit, B. R. T. Trimethylsilyl Derivatives of Organic Compounds in Source Samples and in Atmospheric Fine Particulate Matter. *Environ Sci Technol* **36**, 4273–4281 (2002).

14. Stone, E. A. *et al.* Source apportionment of fine organic aerosol in Mexico City during the MILAGRO experiment 2006. *Atmos Chem Phys* **8**, 1249–1259 (2008).

15. Karthikeyan, S. & Balasubramanian, R. Determination of water-soluble inorganic and organic species in atmospheric fine particulate matter. *Microchemical Journal* **82**, 49–55 (2006).

16. Watson, J. G. Ion chromatography in elemental analysis of airborne particles. (1999).

17. Herner, J. D., Green, P. G. & Kleeman, M. J. Measuring the trace elemental composition of size-resolved airborne particles. *Environ Sci Technol* **40**, 1925–1933 (2006).

18. Lough, G. C. *et al.* Emissions of metals associated with motor vehicle roadways. *Environ Sci Technol* **39**, 826–836 (2005).

19. Environmental Analysis Facility - DRI. https://www.dri.edu/labs/eaf/.

20. Environmental Laboratory (NELAP) Accreditation - Texas Commission on Environmental Quality - www.tceq.texas.gov. https://www.tceq.texas.gov/agency/qa/env_lab_accreditation.html.

21. Alamo, X. X. X., Antonio, S., Alamo, X. X. & Paso, E. *Texas Commission on Environmental Quality*. https://www.tceq.texas.gov/goto/certified_labs.

22. Quality Assurance Guidance Document 2.12 Monitoring PM 2.5 in Ambient Air Using Designated Reference or Class I Equivalent Methods.

23. Landis, E. Quality Assurance Project Plan EPA Category I Filter Handling, Acceptance Testing and Gravimetric Analysis for Chemical Speciation Network, Special Studies and State, Local and Tribal Site PM 2.5 Federal Reference Method Filter Samples. (2018).

24. SUPPORT FOR THE EPA NATIONAL CONTRACT FOR LEAD ANALYSIS.

25. Xiong, Q., Yu, H., Wang, R., Wei, J. & Verma, V. Rethinking Dithiothreitol-Based Particulate Matter Oxidative Potential: Measuring Dithiothreitol Consumption versus Reactive Oxygen Species Generation. *Environ Sci Technol* **51**, 6507–6514 (2017).

26. Yu, H., Puthussery, J. V. & Verma, V. A semi-automated multi-endpoint reactive oxygen species activity analyzer (SAMERA) for measuring the oxidative potential of ambient PM2.5 aqueous extracts. *Aerosol Science and Technology* (2020) doi:10.1080/02786826.2019.1693492.
